# Circadian Rhythms and Epilepsy: A Suitable Case for Absence Epilepsy

**DOI:** 10.3389/fneur.2020.00245

**Published:** 2020-04-28

**Authors:** Magdalena K. Smyk, Gilles van Luijtelaar

**Affiliations:** ^1^Groningen Institute for Evolutionary Life Sciences, University of Groningen, Groningen, Netherlands; ^2^Donders Institute for Brain, Cognition and Behaviour, Radboud University, Nijmegen, Netherlands

**Keywords:** absence epilepsy, circadian rhythms, spike-wave discharges, sleep–wake states, animal models, WAG/Rij rats

## Abstract

Many physiological processes such as sleep, hormonal secretion, or thermoregulation, are expressed as daily rhythms orchestrated by the circadian timing system. A powerful internal clock mechanism ensures proper synchronization of vital functions within an organism on the one hand, and between the organism and the external environment on the other. Some of the pathological processes developing in the brain and body are subjected to circadian modulation as well. Epilepsy is one of the conditions which symptoms often worsen at a very specific time of a day. Variation in peak occurrence depends on the syndrome and localization of the epileptic focus. Moreover, the timing of some types of seizures is closely related to the sleep-wake cycle, one of the most prominent circadian rhythms. This review focuses on childhood absence epilepsy (CAE), a genetic generalized epilepsy syndrome, in which both, the circadian and sleep influences play a significant role in manifestation of symptoms. Human and animal studies report rhythmical occurrence of spike-wave discharges (SWDs), an EEG hallmark of CAE. The endogenous nature of the SWDs rhythm has been confirmed experimentally in a genetic animal model of the disease, rats of the WAG/Rij strain. Well-known detrimental effects of circadian misalignment were demonstrated to impact the severity of ongoing epileptic activity. SWDs are vigilance-dependent in both humans and animal models, occurring most frequently during passive behavioral states and light slow-wave sleep. The relationship with the sleep-wake cycle seems to be bidirectional, while sleep shapes the rhythm of seizures, epileptic phenotype changes sleep architecture. Circadian factors and the sleep-wake states dependency have a potential as add-ons in seizures' forecasting. Stability of the rhythm of recurrent seizures in individual patients has been already used as a variable which refines existing algorithms for seizures' prediction. On the other hand, apart from successful pharmacological approach, circadian hygiene including sufficient sleep and avoidance of internal desynchronization or sleep loss, may be beneficial for patients with epilepsy in everyday management of seizures.

## Introduction

Epilepsy is one of the most common, complex, and multifactorial diseases of the central nervous system. Patients deal with usually unpredictable and often severe seizures, which, if poorly controlled, significantly impact their health, well-being, and quality of life. Although considered as sudden, some types of epileptic activity recur regularly at a particular time of day or during a specific vigilance state. The present review focuses on circadian and also 24-h rhythmicity in seizures' occurrence and the relationship between seizures and the sleep-wake cycle, which itself is a prominent circadian rhythm. Special attention is given to absence epilepsy, a syndrome clearly influenced by these two factors. The circadian timing system is a key, genetically driven mechanism of adaptation to about 24-h environmental cycles. In fact, the majority of physiological processes occur within a circadian range. Similarly, pathological events are very often rhythmic as well. Because general brain activity, neuronal excitability, and various seizures' precipitating factors such as stress hormones follow a circadian pattern, this is also the case for epilepsy in general. Additionally, considering shared thalamo-cortical networks with sleep-wake states, it applies well particularly to absence epilepsy. The following sections give an introduction to the circadian timing system, synthesize information regarding rhythmic occurrence of different kinds of seizures, and focus on absence epilepsy with emphasis on advances in basic research conducted with the use of well-validated animal model, rats of the WAG/Rij strain. Finally, practical implications such as implementation in seizures' predictability algorithms and challenges with respect to, for example, distinction between circadian and sleep influences are discussed.

## Circadian Timing System

The circadian timing system is an evolutionarily conserved mechanism allowing a wide range of organisms (from cyanobacteria to mammals) to anticipate and adapt to cyclic changes in the external environment occurring due to the earth's rotation around its axis (1, 2). From a molecular point of view, it consists of transcriptional–translational feedback loops of core activators, genes, and proteins expressed periodically in practically every cell of the body (3, 4). The system is self-sustained, and as the Latin name indicates (*circa* = about, *dies* = day), it oscillates with a near 24-h period length (5, 6). As every clock, it must be entrained to 24-h day, in this case by environmental cues such as a light–dark cycle, ambient temperature, food availability, presence of predators, or social interactions (7–9). While expression of circadian rhythms in unicellular organisms depends solely on genetic machinery and so-called *Zeitgebers* (German name for a time-giver), in higher plants and animals it requires additionally that two levels of synchronization are achieved: between the organism and the environment, and between single-cell oscillators building structures and organs within the organism (10). In mammals, these tasks are accomplished by a master circadian pacemaker located in the suprachiasmatic nuclei of the hypothalamus (SCN) (11). The SCN receives light signals (one of the most prominent Zeitgebers) directly from melanopsin-containing intrinsically photosensitive retinal ganglion cells via the retinohypothalamic tract (12, 13), integrates the information with other nonphotic time cues (14), generates and imposes rhythm, and synchronizes dependent oscillators in other brain areas and the periphery (15). As a result, circadian rhythms of physiological and behavioral functions are expressed.

## Circadian Rhythms in Disease

There is growing awareness that both internal and external synchronizations are crucial for health and well-being. Circadian disruption caused by, for example, shift work can lead to adverse health consequences and increased risk of cancer, metabolic, neurodegenerative, cardiovascular, or mental diseases (16–19). Very often deviations from well-established rhythmicity (e.g., mistimed episodes of sleep and wakefulness, lack of daily variation in blood pressure, so-called nondipping) are the first signs of serious pathological processes developing in organisms (20, 21). On the other hand, if the acquired pathology regards a rhythmic process or a bodily system that is clearly regulated by the circadian timing system, then it can be expected that the symptoms would follow a circadian pattern as well. Indeed, manifestations of immune system–related diseases, such as rheumatoid arthritis, allergic rhinitis, or bronchial asthma, are most pronounced at night and around awakening, which correlates with a high level of proinflammatory cytokines in the circulation (22, 23). Cardiovascular events such as stroke, myocardial infarction, arrhythmias, and sudden cardiac death are more likely to occur in the morning, which is linked to an increase in sympathetic tone, blood pressure, heart rate, and platelet aggregation (24, 25). Symptoms from a part of the nervous system also follow circadian modulation. Migraine attacks and mood worsening in patients suffering from major depressive disorder occur predominantly in the morning; however, afternoon and evening episodes are also reported (26–28). Exacerbation of behavioral symptoms, so-called sundowning, in some of Alzheimer disease patients takes place during the afternoon and in the evening and is related to a phase delay of the body temperature rhythm (29–31). Epilepsy, conceptually defined as a brain disorder characterized by an enduring predisposition to generate epileptic seizures and their consequences (32), is also a brain disease with a circadian phenotypical expression.

## Circadian Rhythms in Epilepsy

Epilepsy is one of the most common chronic neurological disorders affecting 50 million people worldwide, which accounts for a significant proportion (0.5%) of the global burden of disease (33). Practically, epilepsy is defined as a brain disorder characterized by one of the following conditions: two unprovoked seizures (a transient occurrence of signs and/or symptoms resulting from abnormal excessive or synchronous neuronal activity), which occur more than 24 h apart, one unprovoked seizure and a probability of further seizures similar to the general recurrence risk after two unprovoked seizures occurring over the next 10 years, or diagnosis of any of the epileptic syndromes (32, 34). In general, seizures are classified as of focal, generalized, and unknown onset, with motor and nonmotor subtypes in each of the categories (35). Based on that, the following epilepsy types are distinguished: focal, generalized, combined focal and generalized, and unknown, each of them encompassing a variety of syndromes (36). Epilepsy is a complex disorder of various etiologies (structural, genetic, infectious, metabolic, immune, and unknown), age at onset, underlying neuronal mechanism, clinical manifestation, comorbidities, and therapeutic and prognostic implications. Such a variety of factors may contribute to different circadian patterns of epileptic activity, which were first recognized in patients early in the 19th century (37).

Initial reports classified seizures as diurnal, nocturnal, or diffuse, if they did not show any preference toward the time of a day (38, 39). More recent studies linked a distinct temporal pattern of seizures with a location of the epileptic focus. It was observed that seizures originating in the temporal lobe peaked during the day, whereas those arising from the frontal and parietal lobe were more numerous at night (40–49). Considering that a typical 24-h rest/activity cycle of humans implicates periods of sustained wakefulness during the day and usually consolidated, about 8-h-long sleep during the night, this day/night preference might reflect seizures' susceptibility for a specific state of vigilance. Indeed, sleep and wakefulness, reflecting distinct levels of brain excitability and distinct networks' involvement, are important modulators of the seizure threshold (50). The phenomenon has been implemented in the clinic where sleep deprivation is used to activate epileptic activity in case when a routine diagnostic electroencephalographic (EEG) procedure failed (51, 52). It has been shown that in a group of focal epilepsies temporal and occipital lobe seizures prevailed during wakefulness, whereas frontal lobe seizures during sleep (42, 44, 48, 53–56). Interestingly, this relationship changes with age. In infants, frontal lobe seizures are more prevalent during wakefulness, whereas in adolescents, they are more frequent during sleep (57). Generalized seizures were reported to occur predominantly during wakefulness (46, 47, 58). However, when happening during sleep, they were usually restricted to slow-wave sleep stages I and II; hardly any were recorded during rapid eye movement (REM) sleep (59–64). Also, focal seizures become more often secondarily generalized during sleep (63). Yet, human sleep–wake cycle is one of the most prominent circadian rhythms; besides, temporal organization of sleep depends also on different factors, for example, homeostatic mechanisms. Therefore, a distinction between pure circadian influences and sleep or wakefulness effects without dedicated chronobiological procedures is challenging.

A majority of the studies reporting nonuniform distribution of seizures and/or the relationship between seizures and sleep and wakefulness are based on retrospective analysis of EEG, video EEG, or intracranial recordings performed up to several days for either diagnostic or epileptic surgery purposes. Together with a development of intracranial sensor-based technology for neurostimulation or seizure warning systems, a long-term monitoring (up to even several years) of epileptic activity in nonclinical, patient home settings became possible. Those studies confirmed previous observations regarding a presence of stable 24-h but also longer rhythms in epileptic seizures and interictal epileptiform discharges (IEDs). These rhythms varied with respect to the location of the epileptic focus as well as among individual patients (65–70). Recent studies in humans and in rat models showed stable recurrence of seizures with regard to the phase of the circadian and multidien rhythms pointing toward endogenous mechanisms of such periodicities in epileptic phenomena, coregulation, and interrelationship between seizures and IEDs (67, 71).

## Childhood Absence Epilepsy

Childhood absence epilepsy belongs to a group of genetic generalized epilepsies (GGEs), previously termed idiopathic generalized epilepsies (IGEs) (36). A positive family history is present in 14 to 45% of cases, a concordance of 70% to 85% in monozygotic twins and 30% in first-degree relatives (72, 73). The genotype is complex, including autosomal-dominant gene(s) mutation(s) determining typical EEG trait and channelopathies (74, 75). Childhood absence epilepsy (CAE) is an age-related syndrome with onset between 4 and 10 years, most usually between 5 and 7 years of age, and the annual incidence rate of 6.3 to 8.0 per 100,000 (76–78). It is characterized by frequent (10 to hundreds per day), short (~10 s), typical absence seizures manifested clinically as an abrupt decrease of awareness and responsiveness with cessation of ongoing voluntary activity accompanied by eyes opening, accelerated breathing, and, quite frequently, by automatisms occurring in otherwise healthy children. The ictal EEG is characterized by the presence of 2.5- to 4-Hz bilateral symmetrical synchronized, generalized spike-wave discharges (SWDs) of sudden onset and termination (79, 80). Besides CAE, typical absences are the key symptoms in some other epilepsy syndromes as well, such as juvenile absence epilepsy, juvenile myoclonic epilepsy (JME), and myoclonic absence epilepsy (81, 82). Differences in the age at onset, the frequency and morphology of SWDs, and a prognosis are syndrome-related (80). Proper diagnosis and implementation of pharmacological treatment ensure that 70% to 80% of the patients become seizure-free. Ethosuximide and sodium valproate are established level A medications (efficacious or effective) as an initial monotherapy for children with newly diagnosed or untreated absence seizures (83).

The basic underlying mechanism of generalized absence seizures appears the involvement of the corticothalamocortical neuronal network. Recent studies performed with the use of well-characterized and validated genetic absence animal models revealed that SWDs are generated in the deep layers of the somatosensory cortex (perioral area), which at the beginning of generalization process guide the thalamus and the remainder of the cortex (84–86). Evidence is accumulating that also in humans with absence seizures the initiating side of SWDs is cortical as well (87–90).

Various features of absence seizures, such as neurophysiological single-cell and network interactions and preclinical pharmacological or genetic manipulations, which cannot be investigated in patients because of obvious ethical reasons, are well-modeled in basic research, thanks to availability of the validated animal models. In general, animal models of epilepsy can be divided into two classes: induced, in which seizures are elicited by either chemical or electrical stimulation in naive animals, and genetic (91). Considering chemical stimulation, absence seizures can be induced by a number of agents, such as pentylenetetrazole in low doses, penicillin, γ-hydroxybutyrate, 4,5,6,7-tetrahydroisoxazolo[4,5,-c]pyridine-3-ol, or opiates, administered either systematically or intracerebrally (92–94). Genetic models can also be classified as induced or spontaneous (91). There is a variety of mice strains with a spontaneous single-gene mutation in genes encoding different subunits of high-threshold voltage-dependent calcium channels: lethargic (*Cacnb4*^*Ih*^, lh/lh), stargazer (*Cacng2*^*stg*^, stg/stg), tottering (*Cacna1*^*atg*^, tg/tg), leaner (tgla/tgla), and duky (*Cacna2*^*d*2^, du/du), but also in genes not associated with ion channels: mocha (*Ap3d1*^*mh*2*J*^), coloboma (*Cm, Snap25*), and slow-wave epilepsy mice (*Slc9a1*^swe^) (95). All these strains exhibit SWDs in the EEG; however, their epileptic phenotype is accompanied by severe motor symptoms, such as ataxia or dyskinesia (a result of impaired physiology of cerebellar Purkinje cells), which do not co-occur in human absence epilepsy (95). Considering polygenetic etiology of absence seizures and additional pathological motor symptoms shown by mouse models, the use of two strains of rats: Wistar albino Glaxo from Rijswijk (WAG/Rij) and Genetic Absence Epilepsy Rats from Strasbourg (GAERS), which show spontaneous SWDs concomitant to the mild clinical phenomena without any clear neurological abnormalities, poses higher face, construct, and predictive validity (96–100). Both models are inbred strains originated from Wistar stocks showing similar EEG, behavioral, and pharmacological traits (100). However, as genetic studies revealed, different loci determine the epileptic phenotype in GAERS and WAG/Rij's (98, 99) and therefore may contribute to observed between-strain differences. Moreover, the mutation in Cav3.2 found in GAERS was not present in rats of the WAG/Rij strain (101). Both WAG/Rij rats and GAERS show typical SWDs in the EEG, but while the discharges are longer and more numerous in GAERS, their cycle frequency is slightly higher in WAG/Rij rats (102). Considering spectral characteristics, SWDs of GAERS show greater power in faster components, while preictal period in WAG/Rij rats is characterized by greater power in 8- to 14-Hz frequency band (102), by concomitant delta and theta precursors (103), whereas in GAERS a 5- to 9-Hz rhythm seems to drive the SWDs (104). It should be noted that SWDs are also observed in commonly used, considered as nonepileptic laboratory strains such as Wistar, Sprague–Dawley, Fisher 344, Long-Evans, or Brown Norway rats across their aging process (105–107). However, their epileptic phenotype is less well studied and may differ from that described in GAERS and WAG/Rij rats.

## Circadian Rhythm in the Occurrence of SWDs

**Figure 1:**
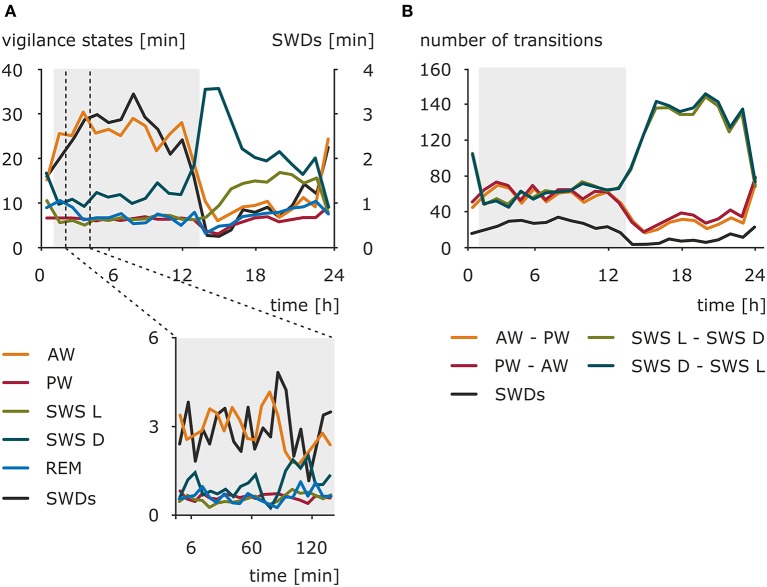
Nonuniform distribution of the SWDs, sleep–wake states, and sleep–wake transitions across 24-h day in WAG/Rij rats, a validated genetic animal model of CAE. **(A)** Total duration (mean) of sleep–wake states and total duration of SWDs in a resolution of 1 h (upper graph) and 6 min (lower graph) (*n* = 12). AW, active wakefulness; PW, passive wakefulness; SWS L, light slow-wave sleep; SWS D, deep slow-wave sleep; REM, REM sleep; SWDs, spike-wave discharges. The dark phase of the 12:12 light–dark cycle is marked by the shaded background. **(B)** Mean of the number of transitions between sleep–wake states across 24 h (data resolution: 1 h). AW–PW, transitions from active to passive wakefulness; PW–AW, transitions from passive to active wakefulness; SWS L–SWS D, transitions from light to deep slow-wave sleep; SWS D–SWS L, transitions from deep to light slow-wave sleep. The dark phase of the 12:12 light–dark cycle is marked by the shaded background. The occurrence of SWDs has a similar course as active wakefulness and opposite to deep slow-wave sleep. The rhythm of absence seizures seems to be coupled to transitions around wakefulness across 24-h day. However, considering slow-wave sleep transitions, phase-related difference might be noticed. The occurrence of SWDs follows them during the dark phase, while in the light phase, seizures are less frequent when the light–deep slow-wave sleep transitions are numerous. Modified from Smyk (118).

Early investigations toward preferable time zones for the occurrence of absence seizures across the 24-h day pointed to the morning as a period with a maximal number of seizures (108–111). Kellaway and colleagues (59) modeled the occurrence of 3-Hz SWDs in a variety of generalized seizures as an interaction between 24-h rhythm and 100-min periodicity of SWDs observed in sleep episodes. The results revealed that the circadian rhythm had a modulatory effect on the amplitude of the discharges peak expectancy during nocturnal sleep (59). More recent studies conducted in a pediatric population and adults found no significant difference in distribution of absence seizures between daytime and nighttime, although in children two peaks were noted: 09:00 to 12:00 and 21:00 to 00:00 (46, 112). Contrary, another study reported a specific pattern of IED distribution in GGE characterized by two peaks (23:00 to 07:00 and 12:00 to 16:00) and two troughs (09:00 to 11:00 and 18:00 to 20:00); however, it should be noted that, apart from typical SWDs, IED spanned also some other epileptiform EEG phenomena (113). These obvious between-studies discrepancies might result from differences in patients' cohorts selected (pediatric vs. adults, single syndrome vs. epilepsy type, pharmacological management of the disease, e.g., successful vs. pharmacoresistance, timing of medication intake), actual variables investigated (seizures vs. IED), and a chosen method of data analysis. Still, it should be kept in mind that in some cases seizures might have a random distribution and do not cluster in a particular time of a day (114) Therefore, the use of standardized, drug-naive, animal models appeared beneficial, also in a process of further validation of the model. The presence of a clear and robust daily rhythm of the number of SWDs was established in WAG/Rij rats in a 48-h EEG recording session. The peak of this rhythm was found in the dark phase, whereas the trough was found at the beginning of the light phase of the 12:12 light–dark cycle (115–117). The distribution of the SWDs across the 24-h day, as well as the amounts of wakefulness (active and passive), light and deep slow-wave sleep, and REM sleep, is presented in Figure 1. However, the duration of the SWDs, ~5 s, did not differ across the 24-h day. The consistency of the circadian rhythm, as established with correlations of the hourly number of SWDs across two 24-h periods, suggested that besides a circadian modulation other factors determine the occurrence of SWDs (115).

**Figure 2:**
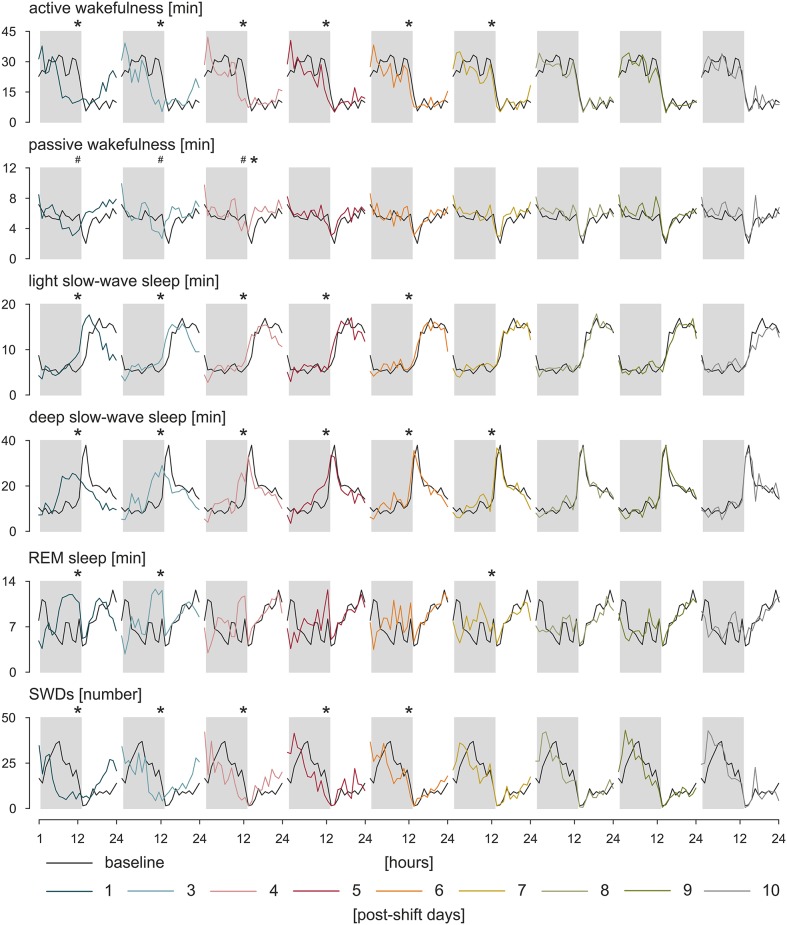
Resynchronization of sleep–wake states and SWDs to an 8-h phase shift in the photoperiod. WAG/Rij rats (*n* = 8) were kept in baseline, 12:12 light–dark cycle. Next, the timing of the light phase was once delayed for 8 h followed by again a 12:12 light–dark cycle. The process of adjustment to the new photoperiod was observed during 10 postshift days. The speed of resynchronization was found rhythm-specific; however, some rhythms, such as active wakefulness and deep slow-wave sleep, and SWDs and light slow-wave sleep resynchronized together, which may indicate that a common circadian mechanism is responsible for those pairs of rhythms. Total duration of sleep–wake states (in minutes) and the number of SWDs during particular postshift days were plotted with respect to the baseline. The dark phase of the 12:12 light–dark cycle is marked by gray rectangles. ^*^p < 0.05, acrophase of the baseline vs. acrophase on the postshift day; #*p* < 0.05, nadir of the baseline vs. nadir of the postshift day, analysis of variance for repeated measures, Bonferroni *post hoc* test or Friedman analysis of variance, and Dunn multiple comparison *post hoc* test (122 with permission from Elsevier).

Circadian rhythms are self-sustained oscillations that persist in the environment lacking time cues (119). Thus, to asses a genuine circadian nature of a given rhythm, subjects must be isolated from Zeitgebers, and further, any variable that might potentially influence the rhythm must be maintained constant. Only one study to date implemented a forced desynchrony protocol, during which all behaviors are evenly scheduled across all circadian phases, and the environmental variables are maintained constant to investigate circadian rhythms of IED in a small subset of patients with JME and IGE (120). Pavlova et al. (120) suggested that there is a clear circadian rhythm of IED independent of any sleep/wake effects; however, because of a small number of participants and high between-subjects variability, the results could not be confirmed statistically. A similar study in search for a true circadian nature of the rhythm of SWDs was performed in WAG/Rij rats (117). Following the approach of constant condition paradigm used previously in a rat model of temporal lobe epilepsy (121), WAG/Rij rats, individually housed, were transferred from the 12:12 light–dark cycle to constant dim light for 20 days, and a comparison was made between the distributions of SWDs across time between baseline and last 7 days spent in constant dim light. In a lack of circadian entrainment, the rhythm of SWDs was still present proving its endogenous origin; however, it desynchronized from the rhythm of general motor activity, which was expressed as opposite regarding changes in period length, mean, and amplitude of the rhythms. It suggested that distinct SCN-dependent oscillators govern these two rhythms. Such an internal desynchronization was accompanied by a somewhat disorganized rhythm of SWDs and an increase in the number of discharges of 45% in the active phase and 17% in the passive phase of the constant dim light, emphasizing a detrimental effect of circadian disruption on the clinical symptoms of the disease (117). Further investigations of the rhythm of SWDs during resynchronization to a new photoperiod after an abrupt, 8-h shift confirmed these findings (122, 123). It took SWD rhythm 6 to 7 days to adapt to the shifted light–dark cycle; the results are presented in Figure 2. During the process, the number of discharges was redistributed between newly established light–dark phases so that SWDs increased in the light and decreased in the dark, and also, they became longer (122, 123). The rhythm of SWDs and light slow-wave sleep shared the same speed of resynchronization different from REM sleep, which appeared to entrain the fastest, whereas the pair of active wakefulness–deep slow-wave sleep was the slowest to re-entrain. It may be assumed therefore that SWDs and light slow-wave sleep are controlled by a common circadian mechanism (123). Importantly, it should be noted that the constant condition protocol used to confirm genuine circadian nature of the rhythm of SWDs controlled the most prominent circadian synchronizer, the light–dark cycle, only. While it proved the endogenous nature of the rhythm of SWDs, it did not resolve the degree to which the shape of the rhythm is influenced by the occurrence of sleep–wake states, which was shown to free-run under constant condition as well (124, 125). The data regarding the resynchronization process of SWDs and the various sleep–wake states are presented in Figure 2.

## Sleep–SWDs Relationship

Absence epilepsy is a network disorder in which pathological oscillations are initiated focally in the cerebral cortex and then propagated and maintained within the corticothalamocortical circuits (98, 126, 127). Thus, not surprisingly, a strong relationship with states of vigilance, which utilize common neuronal substrates, is observed (128). All-night polysomnography revealed that SWDs “prefer” the transitional periods around sleep during which the level of vigilance is low (drowsiness) and progresses into deeper sleep stages, especially stages I and II of slow-wave sleep (129, 130). Typical 3-Hz SWD activity seen in generalized seizures was found greater during nocturnal slow-wave sleep and virtually absent during REM sleep. Three distinct patterns of SWD occurrence within REM/slow-wave sleep cycle were recognized (59). Again, some inconsistencies among reports exist; some studies showed that SWDs occurred predominantly during wakefulness, whereas others pointed to sleep (46, 113, 120). The consensus is made, however, that during wakefulness discharges are activated by somnolence and inhibited by alert wakefulness (131), while during sleep they are restricted to light slow-wave sleep stages and hardly seen during REM sleep (130).

Studies in the genetic models confirmed human data (117, 132–134). Passive wakefulness and light slow-wave sleep reflecting low level of vigilance preceded SWDs most often. Active wakefulness, REM, and deep slow-wave sleep characterized by highly desynchronized (two former) and highly synchronized cortical activity (the latter) had an inhibitory effect on SWDs (117, 132). Experimental procedures aiming at increasing alertness, for example, by engagement in behavioral task reduced SWDs, whereas sleep deprivation known to increase drowsiness had the opposite effect in WAG/Rij rats (135–138). This tight relationship with sleep–wake states, especially with deep slow-wave sleep, is clearly visible in the shape of the circadian rhythm of SWDs. The minimum of the rhythm coincides with the highest amount of deep slow-wave activity during early hours of the light phase (117, 130). Likewise, changes in the number of SWDs during experimental jet lag are well explained by the corresponding changes in the amount of passive wakefulness and deep slow-wave sleep (123). Moreover, although the parameters of circadian rhythm of SWDs changed dramatically under constant dim light condition, the relationships with sleep–wake states described above remained unaffected (117). Thus, it may be hypothesized that, similarly to complementary homeostatic and circadian regulation of sleep, while the generation of SWDs requires a certain state of the neuronal network that is shared with specific sleep–wake states, the circadian timing system provides them with a circadian framework regulating the timing of their occurrence.

**Figure 3:**
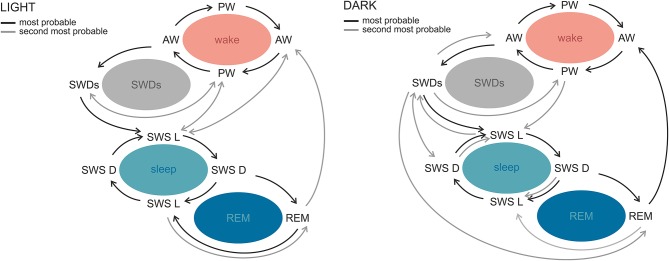
Sleep–wake state transitions in the light and the dark phases of the 12:12 light–dark cycle in WAG/Rij rats. The most stable transitions across the whole sleep–wake cycle and photoperiod were those occurring between active and passive wakefulness and both slow-wave sleep stages. The most prominent phase-related differences regarded SWDs. During the light phase, SWDs were preceded by active and passive wakefulness and followed by light slow-wave sleep or passive wakefulness, while in the dark phase the variability of transitions around SWDs was greater. Similarly, in the light phase, REM sleep was surrounded by slow-wave sleep stages, whereas in the dark phase it was seen most often before active wakefulness. AW, active wakefulness; PW, passive wakefulness; LSWS, light slow-wave sleep; DSWS, deep slow-wave sleep; REM, REM sleep; SWDs, spike-wave discharges (140 with permission from Elsevier).

SWDs occur frequently during sleep–wake transitions both in patients and in the genetic models (129, 130, 133, 138). Albeit rodent sleep is polyphasic, and rats cycle between all sleep–wake stages in practically every hour of 24-h day, clear phase-related differences with respect to total and bouts' durations, as well as number of state transitions, were described (139, 140). A recent study in WAG/Rij rats revealed differential phase-related organization of sleep–wake cycle and transitions around SWDs, which suggests that also this aspect of absence epilepsy might be influenced by the circadian factor (141). In the light phase, SWDs occurred during sleep initiation process: between active and passive wakefulness and before light slow-wave sleep. In the dark phase, however, the variability of transitions from and into SWDs was greater, because discharges occurred before all of the investigated states. Moreover, active wakefulness was seen more often after SWDs in the dark, suggesting that in this particular phase discharges are a part of awaking process; alternatively, SWDs are more susceptible to be aborted by awakenings (141). Figure 3 illustrates the differences of probabilities of transitions between the light and dark phase.

Sleep–epilepsy relationship seems to be bidirectional. Not only do the sleep–wake states modulate seizures' threshold but also epileptic activity of both focal and generalized types impacts sleep. Sleep fragmentation, reduction of various sleep stages, difficulties in sleep initiation, and increased daytime sleepiness are the most common disturbances reported in patients with epilepsy (142–147). On the other hand, no differences in subjectively assessed sleep parameters between patients with well-controlled epilepsy and healthy population were found as well (148). A recently published systematic review on sleep architecture in epilepsy population emphasized a need for well-controlled polysomnographic studies objectively assessing sleep organization in the disorder (149). The review compared 5 of 800 publications that fulfilled strict inclusion criteria and found only one study reporting significantly increased wake after sleep onset in refractory temporal lobe epilepsy in comparison to frontal lobe epilepsy and controls (53, 149–153). There is a multitude of epilepsy-related factors potentially affecting sleep measurements; therefore, a well-defined and validated animal model offers an opportunity of detailed sleep study to be conducted in highly standardized, genetically uniform, drug-naive population. Numerous SWDs in WAG/Rij rats were shown to impact the sleep–wake cycle (154). Two main factors contributed to this effect: circadian, because the sleep–wake cycle and slow-wave sleep were shortened exclusively at the end of the light phase, and the age factor, because such outcome was seen only in older rats that had significantly more SWDs in comparison to their younger intrastrain counterparts (154). The authors concluded that the incidence of SWDs and sleep spindles and the length of the sleep cycle are under genetic control and that the sleep cycle length is also controlled by time of day and age. Non-REM sleep and the sleep cycle were disrupted by the SWDs but only in fragile periods when drowsiness and light slow-wave sleep dominate, and not in the beginning of the sleep period, when rats have predominantly deep slow-wave sleep (154). Moreover, SWDs frequently occurring during the dark phase disturbed a stable slow-wave sleep/REM cycle seen in the light phase (141).

As mentioned before, apart from intended antiepileptic effect, pharmacotherapy of epilepsy may influence sleep parameters as well. It was reported that chronic 4-month treatment with ethosuximide decreased REM sleep duration and REM sleep/total sleep time in WAG/Rij rats; however, the circadian distribution of REM sleep episodes was not affected (155).

## Practical Implications, Challenges, and Further Outlooks

Recognition of temporal organization of epileptic seizures and their dependence on vigilance states possess some promising practical implications for diagnostic, treatment, and prediction purposes. Considering that 30% of patients with epilepsy are refractory including various types of absence epilepsy, there is still quite significant room for improvement of their life quality by means of better epilepsy management. First, sleep deprivation and sleep fragmentation as well-known precipitating factors in some types of seizures are already used in the clinic as a procedure facilitating diagnosis (156). Variation of seizures occurrence across sleep–wake cycle was suggested to be a promising marker in attempts to identify epileptic zone in refractory epilepsy patients evaluated for surgery (157). Moreover, identification of patient-specific rhythms in epileptic activity has been shown to refine algorithms for seizure prediction, which may be subsequently used in seizure warning devices (67, 158). Although a prediction potential of sleep–wake states in absence epilepsy was found to be quite short (4 s) (141), it should not be excluded that the use of such information would increase performance of existing algorithms for SWD prediction (159, 160). Findings regarding circadian disruption caused by either complete lack of entrainment or phase shift (122, 141) point to a risk of worsening of epileptic activity resulting from desynchronization. Possible changes in the timing and duration of sleep–wake states during jet lag should be considered by the patients and physicians while advising before crossing multiple time zones. Additionally, considering close relationship with sleep in this type of epilepsy, proper sleep hygiene, including regularity of sleep schedule and avoidance of sleep deprivation, should be a priority. And this advice is most common given to many people with epilepsy.

Pharmacology is another field of epilepsy management that may benefit from the knowledge of nonrandom distribution of epileptic events. Timed drug administration, one of the principles of chronopharmacology, is already implemented in therapy of, for example, cardiovascular diseases (22). In case of epilepsy, a few studies were conducted on either circadian pharmacokinetics and anticonvulsant action of antiepileptic drugs or the chronotype of patients and its influence on time of medication ingestion (161, 162). However, to fully understand and successfully use, more studies are needed with use of well-validated preclinical models and carefully selected patient populations. Indeed, obtaining homogenous groups of patients for investigations of circadian rhythms in epilepsy might be troublesome. As mentioned before, the majority of studies are retrospective in design. Patients with different epilepsy syndromes (also with wide variety of symptoms within one syndrome), number of seizures, age, age at onset of epilepsy, and different status of pharmacological management of the disease (well controlled vs. refractory, monotherapy vs. polytherapy) were pooled together to form more general groups, for example, “generalized epilepsies” encompassing already distinct syndromes and seizure types, from relatively mild absence seizures to severe tonic–clonic attacks. Such a variety may significantly impact the results of searches for circadian rhythms and clear peaks of seizures occurrence. On the other hand, a certain level of variability in a human population is obviously expected; therefore, long-term studies underlining individual differences in multiple periodicities of recurrent seizures and attempts of practical applications of this knowledge to improve patients' safety and well-being are greatly appreciated.

Moreover, it is also quite challenging, in standard clinical conditions or in patient home settings, to make a sharp distinction between circadian and sleep influences upon observed rhythmicity. According to two process models, sleep is regulated by homeostatic and circadian mechanisms (163). Thus, apart from an accumulating need for sleep, its occurrence is also affected by the circadian timing system. Purely observational studies taking into account states of vigilance around the seizures might be inconclusive to state whether existing rhythmicity in epileptic activity is driven by the endogenous clock itself or indirectly by the sleep–wake cycle, one of the most prominent circadian rhythms. There are validated chronobiological tools such as constant routine or forced desynchrony protocols, separating these two influences (6, 164). However, because of unknown risk for quite a sensitive group of people such as patients with epilepsy and possible hazardous outcomes, they should be implemented with special care.

## Author Contributions

MS and GL contributed toward the preparation, development, and critical review of this manuscript.

## Conflict of Interest

The authors declare that the research was conducted in the absence of any commercial or financial relationships that could be construed as a potential conflict of interest.
